# Soil Microbes and Plant-Associated Microbes in Response to Radioactive Pollution May Indirectly Affect Plants and Insect Herbivores: Evidence for Indirect Field Effects from Chernobyl and Fukushima

**DOI:** 10.3390/microorganisms12020364

**Published:** 2024-02-10

**Authors:** Ko Sakauchi, Joji M. Otaki

**Affiliations:** The BCPH Unit of Molecular Physiology, Department of Chemistry, Biology and Marine Science, Faculty of Science, University of the Ryukyus, Nishihara 903-0213, Okinawa, Japan; yamatoshijimi@sm1044.skr.u-ryukyu.ac.jp

**Keywords:** radioactive pollution, nuclear power plant accident, Chernobyl, Fukushima, soil microbes, plant-associated microbes, field effect, indirect effect

## Abstract

The biological impacts of the nuclear accidents in Chernobyl (1986) and Fukushima (2011) on wildlife have been studied in many organisms over decades, mainly from dosimetric perspectives based on laboratory experiments using indicator species. However, ecological perspectives are required to understand indirect field-specific effects among species, which are difficult to evaluate under dosimetric laboratory conditions. From the viewpoint that microbes play a fundamental role in ecosystem function as decomposers and symbionts for plants, we reviewed studies on microbes inhabiting soil and plants in Chernobyl and Fukushima in an attempt to find supporting evidence for indirect field-specific effects on plants and insect herbivores. Compositional changes in soil microbes associated with decreases in abundance and species diversity were reported, especially in heavily contaminated areas of both Chernobyl and Fukushima, which may accompany explosions of radioresistant species. In Chernobyl, the population size of soil microbes remained low for at least 20 years after the accident, and the abundance of plant-associated microbes, which are related to the growth and defense systems of plants, possibly decreased. These reported changes in microbes likely affect soil conditions and alter plant physiology. These microbe-mediated effects may then indirectly affect insect herbivores through food-mass-mediated, pollen-mediated, and metabolite-mediated interactions. Metabolite-mediated interactions may be a major pathway for ecological impacts at low pollution levels and could explain the decreases in insect herbivores in Fukushima. The present review highlights the importance of the indirect field effects of long-term low-dose radiation exposure under complex field circumstances.

## 1. Introduction

The famous and influential “Atoms for Peace” speech was delivered by the U.S. President Eisenhower in 1953 to promote research and practical applications on nuclear energy and ionizing radiation for benefits to humankind beyond military uses [1]. This speech was delivered 8 years after the speech by the U.S. President Truman to announce the atomic bomb attacks at Hiroshima and Nakagaki, Japan, in 1945 [1], and 58 years after the first discovery of ionizing radiation, X-rays, by Röntgen in 1895 [2,3]. Practical applications of ionizing radiation have indeed flourished in medical technologies (e.g., X-ray examination, computerized tomography scan, and cancer treatment) [4,5,6] and agricultural technologies (e.g., breeding, sterilization of food, and sterilization of insect pests) [7,8]. Basic biological sciences have also advanced [9]. Simultaneously, nuclear fission technologies have culminated in nuclear power plants in addition to ever-powerful nuclear weapons. Unfortunately, not only military uses but also beneficial uses of atomic energy have resulted in serious environmental radioactive pollution due to nuclear bomb tests, radioactive wastes, and nuclear power plant accidents [10].

The massive impacts of radioactive pollution on environments from the beneficial uses were brought about in the Chernobyl nuclear accident in 1986 and the Fukushima nuclear accident in 2011. In both cases, multiple species of radionuclides were dispersed into the surrounding environment [11,12], among which the major pollutant remaining heterogeneously today is radioactive cesium-137 (^137^Cs) due to its relatively long half-life of 30 years [13,14]. In the Fukushima nuclear accident, approximately 300 years are estimated to be required for the current environmental radiation levels to return to pre-accident levels [15]. As such, numerous studies on Chernobyl and Fukushima have investigated the levels of absorbed radiation in humans and other organisms [11,12,13,14,16,17,18]. Although such dosimetric studies are important, they evaluate environmental effects based on estimated doses in reference to laboratory-based irradiation studies alone. Such laboratory studies often use high-level irradiation protocols (e.g., more than 1 Gy in a relatively short time) on the implicit assumption that radiation-induced genetic damage to DNA is the major (and often sole) mechanism of adverse radiation effects. In contrast, many field-based studies [19,20] have focused on population changes alone in the field without any supporting data from laboratory experiments. Alternatively, many field surveys have focused on particular “indicator” species, such as plants, soil invertebrates, insects, and mammals, without much attention to their ecological status. Again, many field-based studies have often been interpreted based on the accumulation of genetic mutations despite relatively low radiation levels compared to laboratory-based irradiation studies.

Without question, these studies provide invaluable information on the biological impacts of the nuclear accidents. However, as these studies accumulate, serious discrepancies in the sensitivity levels of organisms between field data (based on field surveys in contaminated areas or based on field-based experiments) and laboratory data (based on exposure experiments under controlled laboratory conditions) have become evident [21,22,23,24,25]. For example, in the case of the pale grass blue butterfly *Zizeeria maha* in Fukushima, butterfly larvae are vulnerable when they accumulate less than 81.4 Bq/kg (pupa) of radioactive cesium under field conditions [26,27], whereas they appear to tolerate the accumulation of more than 1.62 × 10^7^ Bq/kg (prepupa) under laboratory conditions [28]. In this particular case, the difference in “apparent radioactive tolerance” is extremely large (i.e., more than 2 × 10^5^). Initial acute exposure for the accumulation of genetic mutations is still a valid and important explanation for the high incidence of morphological abnormalities in this butterfly species in the field [21,29,30], but that cannot explain the high vulnerability of the butterfly in Okinawa (not exposed before) to field-collected ^137^Cs-containing host plant leaves [26,27] and the high tolerance of the same butterfly to a ^137^Cs-containing artificial diet [28]. Indeed, in the butterfly, the adverse effects of ingesting contaminated leaves are heritable (transgenerational) but can be cleared if noncontaminated leaves are ingested in subsequent generations [31,32], indicating that the essence of these adverse effects is not genetic changes (involving mutations of DNA bases) but more physiological ones. Such physiological changes may include epigenetic modifications or transgenerational phenotypic plasticity via the maternal effect. Alternatively, continuous exposure to plant toxins over generations may be in effect. Although this butterfly case may be extreme, there may be a possibility that the butterfly case is just the tip of the iceberg: field or laboratory results of other insect herbivores, including Lepidoptera (silkworm moth [33]) and Hemiptera (stink bug [34,35] and aphids [36,37]), seem to be understood similarly. This “field-laboratory paradox” has been a central matter of debate regarding the effects of radiation on animals, including humans [22,23,24,25]. In the case of the Chernobyl nuclear accident, the field–laboratory paradox is likely more complicated than that in the case of the Fukushima nuclear accident because of unclear methodologies, assumptions, and statistical analyses [38].

The field–laboratory paradox appears to be resolved, at least partially, when one considers different mechanisms of biological effects between fields and laboratories [23]. In other words, there is biotic and abiotic complexity in the field and simplicity in the laboratory [23]. Although this point has been emphasized in radioecology since the 1960s [39,40,41,42,43,44], it is still challenging for not only radioactive pollution but also other pollutions, such as those generated by pesticides and heavy metals [45,46]. In the field, there are numerous biotic and abiotic interactions with organisms of interest, which may significantly modify the radiation sensitivity of the organisms to direct exposure. In other words, the synergistic effects of environmental stress and direct irradiation may enlarge the field–laboratory gap. Moreover, organisms in the field may be damaged by other “indirect” mechanisms, which are not necessarily detected in controlled laboratory experiments. For example, changes in plant secondary metabolites may influence plant-eating insects [24]. The bystander effect in fish may also be a good example [47]. More broadly, a nuclear reactor releases not only radioactive substances but also nonradioactive substances, such as particulate matter, which may cause immunological reactions in humans and other animals [48,49]. Immunological reactions to particulate matter are nonexistent in controlled laboratory experiments that consider only radiation levels. Regardless of the degree of radioactivity, these indirect effects may be called “field-specific effects” (or simply, “field effects”) of nuclear power plant accidents [23]. In other words, any indirect changes in the field that are not due to direct radiation exposure but due to the release of radioactive and nonradioactive materials may be collectively called field effects. To add further complexity, the field effect may vary depending on the microenvironment. Depending on the habitat, the ecological half-life (the time required for a radionuclide species to reduce to half of its initial dose of organisms in the habitat, influenced by chemical and biological processes in the ecosystem) of ^137^Cs differs more than 10-fold between organisms that have similar biological half-lives of ^137^Cs [50,51]. The ecological half-life is often longer than the biological half-life and becomes even longer as the rate of decline in radioactive concentration slows over time [52]. As such, numerous mechanisms underlying the biological and ecological impacts of nuclear accidents under complex field conditions may be conjectured [23,46,48,53].

Furthermore, one should admit that there are many unknown indirect pathways for deleterious effects in the field. In that case, these effects cannot be reproduced in dosimetric laboratory experiments simply because they are unknown. These unknown indirect cases were originally emphasized as field effects [23]. Notably, dosimetric explanations implicitly assume that all factors to be controlled in the laboratory are known or that all other factors except radiation dose or dose rate can be negligible when evaluating the biological impacts of a nuclear accident. This assumption is one of the sources of the field–laboratory paradox [23,24,48]. It is essential to recognize that the accumulated knowledge on biological effects in response to nuclear accidents at present is heavily biased toward direct effects and that the standard dose-dependent theory cannot be directly applied to indirect effects. Under conditions of low-dose chronic exposure, indirect effects may have even greater impacts on organisms than direct effects.

Many types of field effects at the individual level likely occur in an ecological context and are often called ecological field effects [23]. It is undoubtedly necessary to position each species-based study in the ecological network. The importance of ecological producers (i.e., photosynthetic plants) cannot be overemphasized to understand an ecological network in a contaminated environment. Herbivorous animals are completely dependent on food supplies from plants as ecological consumers. Most plants depend on soil for survival, and soil is the product of soil microbes. Soil microbes decompose organic materials and make nutrients useable for plants. Moreover, plants often harbor plant-associated microbes as symbionts, mainly bacteria and fungi, and they improve plant growth and pathogen resistance, which determines food quality for herbivorous insects. Therefore, we speculate that microbe-plant interactions may be the key to understanding many unknown aspects of the field effects on plants and insect herbivores.

To examine the possibility that microbes are the key to understanding the field effects on plants and insect herbivores, the present review focuses on field-based surveys, with some exceptions, on soil microbes and plant-associated microbes (mainly bacteria and fungi but also soil invertebrates, i.e., nematodes and earthworms) in radioactively contaminated environments in Chernobyl and Fukushima, interpreting the effects on plants and insect herbivores through the food web. We believe that such microbe-mediated effects are largely underestimated at present because evaluations are often exclusively based on dosimetry and laboratory experiments rather than field effects. It should be mentioned that radiation sensitivity is not the same between the field and laboratory; organisms in the field are said to be eight times more sensitive, as first statistically presented in Chernobyl research [22]. Thus, in this review, irradiation experiments were excluded unless otherwise noted. When Sievert (Sv) was used as a unit of radiation dose in a paper, it was converted to Gray (Gy) (1 Sv = 1 Gy).

## 2. Multiple Pathways for Biological Effects

**Figure 1:**
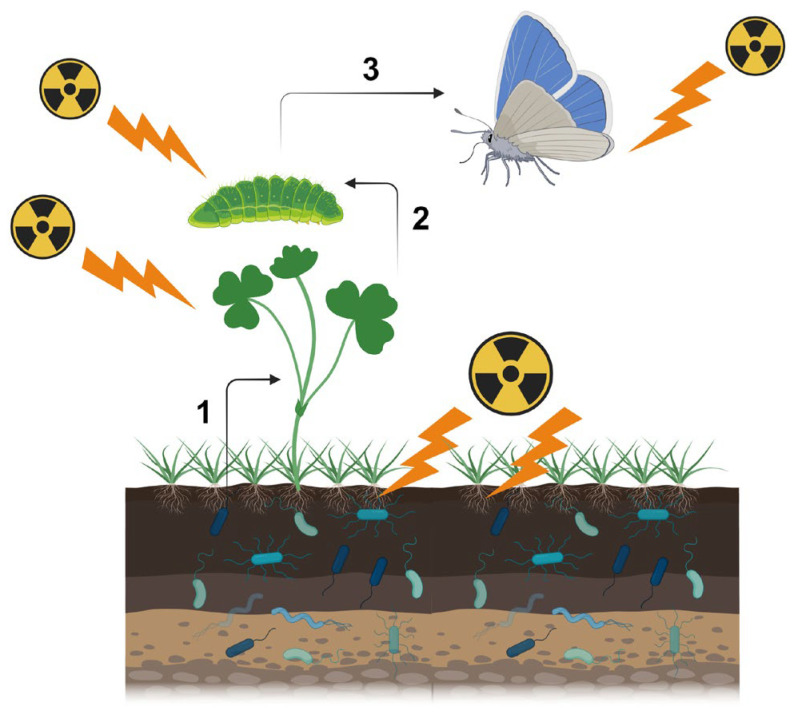
Direct and indirect effects of a nuclear accident on insect herbivores in the context of the ecosystem. Here, the butterfly system is depicted as an example. All entities (soil microbes, plant-associated microbes, plants, larvae, and adult butterflies) are irradiated in radioactively contaminated areas simultaneously, but because radioactive materials accumulate in soil, microbes may be affected the most in terms of exposure levels. Changes in microbes will affect plants (microbe-mediated effect indicated as “1”), and changes in plants will affect plant-feeding larvae (metabolite-mediated effect indicated as “2”). Adult butterflies are then affected at the population level (indicated as “3”). In this figure, plant-associated microbes are not shown. This figure was created with BioRender.com and Adobe Photoshop Elements.

First, one should recognize that there are multiple pathways for biological effects of a nuclear accident. Here, one should be careful about the terminology. At the molecular level, “direct” damage occurs when ionizing radiation directly breaks the covalent bonds of biological molecules (especially those of DNA), and “indirect” damage occurs via reactive oxygen species (ROS) produced by water radiolysis [23]. At the individual (organismal) level, both direct and indirect molecular mechanisms for ionization are considered “direct” effects on a given organism. As a secondary effect of these “direct” effects, several “indirect” effects may occur. For example, the sole host plant of the pale grass blue butterfly may synthesize insect toxins in response to irradiation, and butterfly larvae die due to ingested toxins (Figure 1). Changes in metabolites in the host plant may be considered direct or primary effects, and if so, larval death is the secondary effect. However, the primary and secondary effects may not always be fixed. Plant metabolite changes may be caused by soil microbes and plant-associated microbes, and if so, these microbial changes are the primary effects; plant metabolite changes should be considered secondary effects, and larval death should be considered to occur through tertiary effects. In reality, soil microbes, plant-associated microbes, plants, and insects are exposed to radiation simultaneously. That is, these direct effects and indirect field effects work simultaneously in the field.

In addition to the direct and indirect effects discussed above, these effects may result in population-level changes in the numbers of animal species in the field. When a key species in a food web is highly sensitive to radioactive pollution, the balance of the food web may be disrupted, and other species may be affected “indirectly” due to a lack (or surplus) of food. This type of population-level indirect effect is theoretically straightforward and does not always lead to a paradoxical interpretation against laboratory-based results, especially at high doses that directly eradicate a given species. The population sizes and ^137^Cs concentrations of many organisms (including spiders [54,55], cicadas [54], dragonflies [54,55], butterflies [54,55], grasshoppers [54,55], bark beetles [17,55], bumblebees [54,55,56], booklice [17], springtails [17], soil invertebrates [57,58], reptiles [56], birds [54,59], and mammals [56]) have been reported in field surveys in Chernobyl. Some of these population changes have been discussed in terms of their predator–prey relationships. A decrease in the number of organisms attributed to food loss is an indirect consequence of radiation exposure of the ecological community [17,18] (see also Section 5.1).

**Figure 2:**
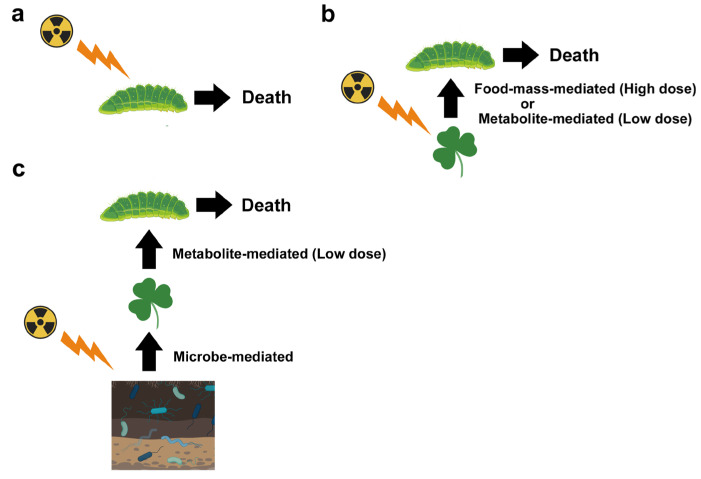
Different pathways that could lead to the death of insect herbivores (butterfly larvae as an example) in radioactively contaminated fields. (**a**) Direct effect only. Most dosimetric studies take this standpoint. If larvae are affected directly by high-dose exposure, this type of study may be consistent with a decrease in butterfly (adult) populations. (**b**) Larvae receiving the secondary effect from their host plant leaves. After high-dose exposure, plants may die, resulting in larval death due to a lack of food (food-mass-mediated effect). After low-dose exposure, plants may synthesize secondary metabolites that are toxic to larvae, resulting in larval death due to the toxins (metabolite-mediated effect). (**c**) Larvae receiving tertiary effects from their host plant leaves (metabolite-mediated effect) that receive secondary effects from soil microbes and plant-associated microbes (microbe-mediated effect). Depending on the exposure level, microbes, and other soil conditions, the effects on plants may vary and be nonlinear. In the field, synergistic effects from other stressors may also occur, but they are not indicated in this figure. This figure was created with BioRender.com and Adobe Photoshop Elements.

The distinction between direct and indirect effects and the distinction among primary, secondary, and tertiary effects require careful and laborious laboratory experiments that consider field conditions. However, researchers should consider these potential effects carefully to interpret field data. It is important to note that the same biological result (e.g., the death of butterfly larvae) may be caused by very different mechanisms (Figure 2). Therefore, one study may necessarily focus on a small system but should clarify the scope coverage (i.e., which potential species interactions are considered or not) when presenting field or laboratory data. Unfortunately, many studies do not seem to recognize the importance of indirect effects, resulting in inaccurate estimates and interpretations of the biological and ecological impacts of nuclear accidents.

## 3. Soil Microbes and Soil Invertebrates

### 3.1. Chernobyl Studies

Field-based surveys of soil microbes began shortly after the Chernobyl accident in April 1986 to observe microbial abundance, species diversity, and composition. One of the earliest studies, from 1986 to 1989, within the 30 km zone of the Chernobyl nuclear power plant (NPP), called the exclusion zone, found radioresistant mycobiota and discovered an increase in melanized mycelia [60,61], whose function has been extensively investigated since the 2000s [62,63,64]. During this time period (1986–1989), rainfall transferred radionuclides from the plant surface to the soil [13], and most radiation doses were due to β-rays [17], which had a large impact on soil microbes. In the 1990s, several papers reported the reduced abundance and species diversity of soil microbes [61]. The number of heterotrophic bacteria and the total bacterial species in the surface layer (0–20 mm depth) of the 10 km zone of the NPP were less than those in the control site, indicating that an altered population size had not yet recovered by 1993 [61,65]. Aerobic chemoorganotrophic bacteria were cultured from the soil of the 10 km zone on nutrient agar, and the abundance of cellulose-fermenting, nitrifying, sulfate-reducing, and nitrogen-fixing bacteria, as well as heterotrophic ion-oxidizing bacteria, decreased by up to two orders of magnitude in number [61,66].

Similar declines in abundance and species diversity are known for soil invertebrates [67]. These changes in numbers are understandable considering their food web interactions. Lecomte-Pradines et al. (2014) [58] noted that radiation might have indirectly affected the abundance of nematode assemblages collected within 30 km of the Chernobyl NPP in 2011 by modifying their food resources, i.e., soil bacteria (see also Section 5.1). Moreover, 11 mGy/h (23 Gy in total) of chronic irradiation with ^60^Co, which is approximately 1/30 of the LD_50/30_ (half lethal dose for 30 days), or 650 Gy for acute irradiation with ^137^Cs induced severe adverse effects on the reproduction of adult earthworms [68,69]. Thus, the reduced population of soil invertebrates (nematodes and earthworms) could be caused by both direct irradiation and an indirect lack of food. According to the International Atomic Energy Agency (IAEA) (2006) [13], within two months of the accident, the number of invertebrates in the litter layer of forests became 1/30 (3–7 km from the Chernobyl NPP), and the estimated cumulative exposure amount at that time was 30 Gy.

In the 2000s, research methods and technology drastically improved as metagenomic analysis emerged to address a large number of output data [70,71]. Regarding abundance, Beresford et al. (2022) [72] measured the feeding activity of soil fauna via the bait-lamina strip test and demonstrated that biological activity was low in “the Red Forest” in the 10 km zone of the Chernobyl NPP, in 2016, due to a residual effect of acute exposure just after the accident. This finding is consistent with that of Mousseau et al. (2014) [73], which showed that leaf litter mass loss was 40% lower at the most contaminated site within 30 km of the Chernobyl NPP in 2007 than at the site with a normal background radiation level. Based on these studies, it is likely that the soil microbial and invertebrate populations remained less abundant in highly contaminated areas even more than 20 years after the accident. This conclusion is also consistent with the findings of the older studies in the 1990s discussed above [62,66].

Conversely, Bonzom et al. (2016) [74] experimentally reported no detrimental effects on the decomposition of leaf litter in 2010, indicating that microbial functions and populations are likely normal. This contrasts with the findings of Mousseau et al. (2014) [73] discussed above. Interestingly, both surveys [73,74] were carried out around the same time in the exclusion zone and considered potential confounding factors, such as pH and soil moisture. One of the reasons for these contrasting results may be the difference in radiation dose rate on the ground: 240 μGy/h in Mousseau et al. (2014) [73] and 29 μGy/h at most in Bonzom et al. (2016) [74].

Videvall et al. (2023) [75] indicated that the microbial composition of soil in wetlands collected in 2019 was affected by radiation, and several microbial taxa, including radioresistant bacteria, were more abundant within 30 km of the Chernobyl NPP. On the other hand, Chapon et al. (2012) [76] concluded that there was no high selection pressure on bacterial communities 2.5 km from the Chernobyl NPP, called trench 22 [77], in 2008 and 2009, which contained clean-up waste from the Red Forest. The discrepancy between the findings of Videvall et al. (2023) [74] and Chapon et al. (2012) [76], both of which used the same method of 16S rRNA gene sequencing, could be due to differences in the radiation dose rate or soil type at the experimental sites (wetland and sandy soil of trench 22). These two papers disagreed on the composition but agreed on the species diversity; they found rich and diverse microbiomes. This finding is also supported by the study conducted by Ruban et al. (2020) [78], which demonstrated that the largest number of species were identified in the most contaminated site, the Red Forest, based on the α diversity index. The increase in species diversity reported in these relatively recent studies contrasts with the decrease in species diversity reported in earlier studies in the 1990s [62,66].

The air radiation dose rate was measured in October 1987, 1.5 years after the accident, and reached 0.10 mGy/h at the 4.0 km site and 0.35 μGy/h at the 16.0 km site from the reactor in Chernobyl [13]. Thus, the dose rate was very different even inside of the exclusion zone within 30 km, where most of the surveys reviewed in this section were conducted. Depending on the radiation dose, not only the acute impact on microbes but also the process of recovery from radiation (more precisely, to reach a stable state) may differ. Therefore, some seemingly contradictory results discussed above are not necessarily contradictory. Shuryak et al. (2016) [79] suggested the occurrence of a nonmonotonic dose response during the growth of microbes: stimulation at low concentrations and inhibition at higher concentrations.

There is possibly a threshold in radiation dose, i.e., a boundary between the presence or absence of initial exposure for organisms to have negative effects. The Chernobyl Forum organized by the IAEA officially summarized two decades of research and established three phases of radiation exposure: (i) the first 20 days, (ii) from summer to autumn in 1986, and (iii) from the beginning of 1987 to the present [13]. The total cumulative dose at the end of the third month after the accident, between the first and second phases, was estimated to be 0.7 Gy by the thermoluminescent dosimeters on the soil surface within 30 km from the Chernobyl NPP [13]. Most of the studies mentioned in this section were carried out in the third phase, where the dose rate was less than 1% of the initial value, and the amounts of β- and γ-radiation became comparable. In this phase, two-layer exposure must be considered: initial acute exposure to approximately 25 radionuclides with various half-lives and ongoing chronic exposure to much fewer radionuclides with long half-lives. The initial exposure was intensive and much greater than the subsequent chronic exposure. The most concerning radionuclides are ^131^I (for initial acute exposure) and ^137^Cs (for chronic exposure). Depending on the initial exposure levels, the output data may vary.

The radioresistance of microbes in soil [62,80,81] is known to rely on powerful DNA repair mechanisms [62,82,83]. *Cladosporium sphaerospermum*, which is a “radiotrophic fungus” that utilizes melanin for energy metabolism [84], is expected to serve as a radiation shield to protect humans from space radiation and was sent to the International Space Station for cultivation experiments [85]. Some bacteria and fungi, such as *Agrobacterium* sp., *Enterobacter* sp., *Klebsiella* sp., and the family Cortinariaceae, can accumulate ^137^Cs at high concentrations [62,86]. Enzymes in these decomposers play key roles in the transfer and cycling of ^137^Cs in ecosystems [87,88,89]. In fact, Rufferty et al. (1997) [90] explained that the decomposition of litter in forests was accompanied by an increase in ^137^Cs, which was attributed to the importation of decomposer fungi. Zhdanova et al. (2004) [80] demonstrated that approximately 200 species of fungi have been isolated from the Chernobyl NPP, and most of them can grow with hot particles. According to Jonsson et al. (1999) [52] and Wada (2021) [91], the ecological half-life of ^137^Cs in the Arctic charr fish *Salvelinnus alpinus* in a lake contaminated by the Chernobyl fallout was 1.5 years based on the first 4 years but 22.4 years based on 12 years after the accident.

### 3.2. Fukushima Studies

The Fukushima nuclear accident occurred in March 2011, 25 years after the Chernobyl nuclear accident. Although advanced research methods and techniques have become more accessible, only a handful of studies on soil microbes have been documented in Fukushima. Ihara et al. (2021) [92] explored the soil bacterial community at the base of mugwort via high-throughput sequencing. They approached 1 km to the NPP at the closest location, where the ^137^Cs concentration in the soil sample was 563 kBq/kg (dry) in 2014. Notably, for comparison, soil samples were collected from four geographically remote sites with the same vegetation and land use. The authors demonstrated the following three points in terms of bacterial communities: at the most contaminated site, (i) the species diversity was lower, (ii) the composition was different, and (iii) the radioresistant bacterium *Geodermatophilus bullaregiensis* was more abundant. Similarly, Higo et al. (2019) [93] examined the community dynamics of the arbuscular mycorrhizal fungus colonizing the roots of napiergrass *Pennisetum purpureum* under different land uses (paddy field and grassland) before an accident within 30 km of the Fukushima NPP in 2013 and 2014. The deposition density of ^137^Cs was 3404 kBq/m^2^ in paddy fields and 3322 kBq/m^2^ in grasslands at the time of 2013. Illumina MiSeq sequencing data revealed that species diversity was lower in 2014 for both land-use types and that the species composition differed between sampling years and between land-use types. The most abundant family, Glomeraceae, may be tolerant of complex environments [94,95]. In addition, phospholipid fatty acid (PLFA) analysis has been widely used to estimate the total biomass and composition of microbes. Huang et al. (2016) [96] reported that fungal biomass, but not bacterial biomass, was positively correlated with the ^137^Cs concentration in the litter of deciduous forests 50 km from the Fukushima NPP during the first year after the accident. Additionally, the ^137^Cs concentration in the leaf litter increased from 3290 Bq/kg to 29,800 Bq/kg during the 10-month experimental period through a decomposition process [96]. The three studies [92,93,96] ranged from 1 km to 50 km from the Fukushima NPP for a limited 4 years after the accident, with gradual dose effects.

To the best of our knowledge, there are no reports on the population or species diversity of soil invertebrates in Fukushima, despite many reports on radioactivity concentration measurements. According to Garnier-Laplace et al. (2011) [97], the dose rate of the sum of ^131^I, ^134^Cs, and ^137^Cs for one month after the accident was calculated to be 2.3 mGy/day for soil invertebrates using the ERICA assessment tool based on the soil sample of Iitate Village, which was much less contaminated than that in Chernobyl. However, since the Chernobyl accident, the uptake of ^137^Cs by fungi has been well known, and mushrooms that exceeded the standard limit for general foods set by the Japanese government were found as early as the autumn of 2011 [98,99].

### 3.3. Commonalities between Chernobyl and Fukushima

Declines in the abundance and species diversity and compositional differences in soil microbes have been reported both in Chernobyl and Fukushima, and some microbes with radioresistance or accumulation of ^137^Cs have been reported in both studies; however, in highly contaminated areas in Chernobyl, composition and species diversity may not follow these rules. Furthermore, the ^137^Cs concentration in leaf litter increased during the decomposition process in Chernobyl and Fukushima, and the movement of ^137^Cs in soil was potentially mediated by microbes to organisms in the soil and on the ground through trophic connections. As ^137^Cs is cycled and maintained in the environment over time, the ecological half-life becomes much longer than initially estimated.

Species diversity is understood to decrease due to the simultaneous elimination of more radiosensitive species and also due to the increase in radioresistant species and immigrants [17]. In other words, radioresistance is a key trait for the abundance, species diversity, and composition of overall microbial communities in soil. Additionally, in general, radioresistant bacteria are resistant to ultraviolet rays [100,101] and dryness [102], suggesting that soil microbes adapt flexibly to various environmental stressors. Therefore, the abundance, species diversity, and composition of radioresistant microbes in the field seem to fluctuate on a large scale in response to stressor types. Barescut et al. (2011) [103] suggested that disturbances in ecological interactions are caused by changes in biological pressure resulting from species differences in radioresistance and ecologically open niches for the immigration of new species.

Here, another example of a radioactively exposed field is given. Chengdu in China was contaminated by low-dose ^232^Th for more than 10 years, and Cheng et al. (2023) [104] investigated the diversity and composition of microbes in 20 soil samples of which soil type, moisture, and pH did not differ. They found that fungal diversity was significantly reduced in irradiated soil groups (absorption dose rate of γ-rays: 630 nGy/h and 840 nGy/h) but not in bacterial diversity and that the compositions of both fungal and bacterial communities were affected. Bacteria could be more adaptive than fungi in general. In fact, proteobacteria exhibiting strong adaptability predominate in soil exposed to radiation and maintain the stability of the bacterial community [104]. These findings suggest that differences in radioadaptability could drive imbalances at any taxonomic level from domain to species in the soil ecosystem.

Chernobyl and Fukushima are two unique field study sites with natural settings. Comparisons between these factors in terms of not only radiation dose rates and radionuclide species but also other environmental factors, including soil type and vegetation, could be used to assess the impact of nuclear accidents on soil ecosystems. The most recently published paper reviewed in this section is Videvall et al. (2023) [75], which mentioned “we still know very little” about microbes in the environment of Chernobyl. This is also true in Fukushima. Therefore, there is much room for new discovery on soil-microbe-mediated effects on plants both in Chernobyl and Fukushima.

## 4. Plant-Associated Microbes

### 4.1. Chernobyl Studies

Studies on herbaceous plants were initiated as early as 1986 (see also Section 5). Geras’kin et al. (2002) [105] collected seeds of winter rye within 30 km of the Chernobyl NPP approximately 4 months after the accident, and after germination, the plants were subjected to cytogenetic tests. In a different study, germination of wild carrot seeds from maternal plants exposed to radiation in Chernobyl showed the lower gemination rate and other abnormal life-history traits [106]. However, plant-associated microbes have rarely been explored. Mousseau et al. (2014) [73] suggested that the reduced rate of litter mass loss and thicker forest floor (poor levels of decomposition in other words) in the 30 km zone of Chernobyl could have an effect on growth conditions for plants because free-living microbes strongly regulate plant productivity through mineralization during the decomposition process, which makes nutrients such as nitrogen and phosphorus available to plants [107]. Although the following study is not the case for Chernobyl, in halophytes grown in radioactively contaminated fields with ^137^Cs, Zhu et al. (2021) [108] showed that the richness of fungal, not bacterial, communities in plant roots was positively correlated with the total amount of nitrogen in soil but was somehow negatively correlated with organic matter. That is, decreases in soil microbes, decomposed soil, and consequently nitrogen in soil may indirectly lead to a decreased population of plant-associated microbes and developmental aberrations in plants. Numerous studies have shown that plant-associated microbes play a significant role in plant growth [109,110,111,112,113]. Among them, arbuscular mycorrhizal fungi (AMF) and plant growth-promoting rhizobacteria (PGPR) are well known for fixing nitrogen.

Several papers have noted that the effect of radiation exposure on plants is a weakened defense system when radiation levels are relatively high. A decrease in the disease resistance of wheat, rye, and maize was observed within 10 km of the NPP, and in fact, brown rust and true mildew infection increased in winter wheat, corresponding to radioactive contamination [11]. Simultaneously, the emergence of a new causal agent of stem rust, *Puccinia graminis*, with a high frequency of more virulent clones was detected within 10 km [11,114]. Thus, the prevalence of plant diseases in Chernobyl could be caused by both reduced disease resistance and enhanced toxicity.

In addition to those in the Red Forest, morphological and biochemical changes in plants in response to high acute radiation exposure have been observed, regardless of the involvement of plant-associated microbes [115]. However, the radiation sensitivity of plants varies depending on the species, developmental stage, genome size, and photosynthetic activity [116,117]. For example, oxidative and genetic damage to Scots pines has been reported even at low radiation levels [118]. Importantly, plant growth, development, and stress tolerance are promoted at low exposure levels, which is known as radiation hormesis [119]. Gudkov et al. (2011) [120] reported that biochemical changes began to occur at 10 μGy/h or less based on studies of the Chernobyl, East-Ural radioactive trace, and Semipalatinsk nuclear test site. Similarly, radiation exposure may enhance the defense system, including the upregulation of antioxidants and insect toxins, in the case of low chronic radiation exposure; crucially, such a process may be promoted by mutualistic microbes [121,122]. Other abiotic stresses may similarly promote the plant defense system [123,124].

### 4.2. Fukushima Studies

Within the context of even fewer studies on plant-associated microbes in Fukushima, Sakauchi et al. (2022) [125] subjected the field-picked creeping wood sorrel *Oxalis corniculata* to LC–MS analysis to quantify secondary metabolites. The radiation level ranged from nondetectable to 718 Bq/kg for the ^137^Cs radioactivity concentration in the leaves and from 0.04 μGy/h to 4.55 μGy/h for the ground dose rate at which the leaves grew. This study demonstrated that *Oxalis* leaves, which were field-picked in Fukushima and looked completely healthy to the naked eye, upregulated and downregulated secondary metabolites in response to low-dose radiation exposure [125]. Seven compounds with single annotations were microbe-derived out of twenty-two, four of which were antibiotics produced by *Streptomyces* sp., a major group of soil bacteria, and by actinomyces that can also be isolated from *Oxalis* [126,127]. Half of the four antibiotics were upregulated, and the other half were downregulated in a dose-dependent manner. A logarithmic model fit better than a linear model for three of the antibiotics, indicating a nonmonotonic radiation stress response in *Streptomyces*.

In addition to these findings, Zhu et al. (2021) [108] studied contaminated soil samples containing three different ^137^Cs concentrations (low: 20–40 Bq/kg, medium: 40–60 Bq/kg, and high: >60 Bq/kg) from a historic nuclear test site in China and found that the richness of the endophytic bacteria in the roots of *Kalidium schrenkianum* was significantly greater only in low-radiation soil than in the control soil. Thus, endophytes could sensitively change their abundance in response to low-radiation exposure. Sakauchi et al. (2021) [128] also performed LC-MS analysis on irradiated *Oxalis* leaves in a laboratory using radioactively contaminated soil collected from Fukushima (34 μGy/h for seven days) as a radiation source, but no common compound was upregulated or downregulated between field-polluted and laboratory-irradiated leaves, which might be attributable to differences in radiation exposure conditions.

From the viewpoint of agricultural interest, Aung et al. (2015) [129] inoculated *Bacillus pumilus*, a plant growth-promoting rhizobacterium (PGPR), into four vegetables from the Brassicaceae family growing in Fukushima and evaluated its growth promotion and ability to absorb ^137^Cs. Consequently, inoculation did not enhance plant growth but increased both the accumulation of ^137^Cs in plants and the speed at which ^137^Cs was transferred from the soil to plants. In addition, in *Streptomyces* sp., K^+^ channels did not efficiently distinguish Cs^+^ from K^+^, both of which are alkali metal ions [130].

### 4.3. Commonalities between Chernobyl and Fukushima

In Chernobyl, various adverse effects have been observed on plants, including morphological changes, disturbances in growth, suppressed reproductive ability, death, disease, and pest infections [13]. A few of these events have also been observed in Fukushima [131,132,133,134]. It is highly likely that plant-associated microbes are involved in these observations, although no causal relationship has been demonstrated thus far. No reports of poor growth, disease, or pest infections were available in Fukushima, despite the large number of crop fields and fruit trees in the contaminated area.

Partially considering Mousseau et al. (2014) [73] and Zhu et al. (2021) [108], plant-associated microbes may become less common in relatively severely contaminated areas in Chernobyl and Fukushima, possibly leading to growth failure and low immunity in plants. For example, arbuscular mycorrhizal fungi (AMF) that colonize plant roots and form symbiotic associations with 80% of terrestrial plant species are generally accepted to contribute to plant growth by facilitating the production of growth hormones and phosphorus uptake. The antibacterial endophytic fungus *Streptomyces galbus* improved resistance to Pestalotia disease, root rot, and anthracnose and was inoculated for practical use on flowering plants such as *Rhododendron* [135]. On the other hand, based on the LC–MS analysis of *Oxalis* leaves in Fukushima, the abundance of *Streptomyces* sp., which produces antibiotics, did not always decrease [125]. In Cheng et al. (2023) [104], ectomycorrhizal fungi in soil irradiated by low-dose ^232^Th for more than 10 years were positively correlated with radiation intensity. The hyphae of these fungi wrap around plant root cells and form a biomembrane that not only protects plants from pathogens, chemical pollutants [136,137], and radiation but also helps plants absorb soil nutrients, including nitrogen and phosphorus [138]. Therefore, it is quite conceivable that the growth and defense system of plants are influenced by increasing or decreasing the abundance of plant-associated microbes or the emergence of more toxic pathogens in Chernobyl and Fukushima. In addition, endophytes are transmitted vertically via seeds or pollens and horizontally via the soil or atmosphere [139,140]. Thus, radioactively contaminated environments might have naturally affected both transmission processes.

Considering that the targets of plant species and chemical components differ between Sakauchi et al. (2022) [125] and Gudkov et al. (2011) [120], 10 μGy/h may be appropriate as the rough minimum dose rate for biochemical changes, including changes in endophyte status, as the mildest effect. Similarly, ultraviolet-B irradiation (13.3 kJ m^2^/day), corresponding to 25% of ozone depletion in mid-summer in New Zealand, induced foliar chemistry changes in the white clover *Trifolium repens* [141].

Here, it should be emphasized that the status of plant-associated microbes can fluctuate due to biotic and abiotic factors. U’Ren et al. (2012) [142] concluded that climatic patterns, geographic separation, host type, and host lineage affect the abundance, diversity, and composition of plant-associated microbes. According to Rilling et al. (1999) [143] and Sanders et al. (1998) [144], the presence of external and internal hyphae of arbuscular mycorrhizal fungi (AMF) increase as a result of elevated atmospheric carbon dioxide. In the case of plant growth-promoting rhizobacteria (PGPR), drought conditions may promote cellular division in roots and root hairs [145]. Both elevated carbon dioxide and drought result in the enhancement of water and nutrient uptake and lead to the improved growth of host plants. Similarly, radiation stress likely stimulates the potential ability of plant-associated microbes to cause host plants to grow and resist diseases and pests. Sufficient consideration of any environmental factor is required even when radiation is targeted, keeping in mind the nonmonotonic stress response.

## 5. Plants and Insect Herbivores

### 5.1. Food-Mass-Mediated Indirect Effects

Early studies, mostly conducted in 1986 at the time of the Chernobyl accident, reported reproductive degradation in various herbaceous plants [11]: a reduced number of seeds or a lower germination rate in winter wheat, cocksfoot *Dactylis glomerata*, and ribwort plantain *Plantago lanceolata*, and sterility in winter wheat, winter ryes, and wild vetch *Vicia cracca*. Taskaev et al. (1992) [146] observed no effect on the seeds of 15 species within 30 km of the Chernobyl NPP. Boratyński et al. (2016) [106] conducted a germination experiment using seeds of the wild carrot *Daucus carota,* collected from an abandoned field within 10 km from the Chernobyl NPP in 2012, and showed that the more radiation the maternal plants were exposed to, the longer the time that the seeds took to germinate and produce leaves and the lower the germination rate. Therefore, it is reasonable to speculate that the overall mass of phytocoenoses decreased around the Chernobyl NPP in heavily contaminated areas. Indeed, according to Suvorova et al. (1993) [147] (cited in Geras’kin et al. (2008) [11]), the total number of plants was reduced from 740 to 310 specimens/m^2^, and the number of species per 100 m^2^ became approximately one-fourth simultaneously, with less species diversity two years after the Chernobyl accident. Moreover, the abundances of several species, such as heather *Calluna vulgaris*, increased following interspecies competition against radiosensitive species [11]. Although the underlying mechanism is not known, some of these negative effects might be attributed to plant-associated microbes, which influence plant growth or pathogen invasion (see also Section 4). However, many of the changes in the number of plants discussed above are likely caused by direct effects when the radiation level is relatively high. Alexakhin et al. (2004) [148] (cited in Geras’kin et al. (2008) [11]) estimated that the radiation dose to plants within 30 km of the Chernobyl NPP was sufficient to induce mortality, sterility, and a reduction in the productivity of some species. This is perhaps the reason why there have been no reports on changes in the population or diversity of herbaceous plants in Fukushima, where the contamination level is relatively low.

The perturbation of phytocoenoses causes severe impacts on insect herbivores, which have no other option but to eat plants. Generalist herbivores may converge on surviving radioresistant plants. As a result, interspecies competition necessarily becomes more intense. In the case of specialist herbivores, survival will be difficult if their host plants are sensitive to radiation. This indirect effect through food loss, which many be called the food-mass-mediated effect, was mentioned in the early 1970s based on irradiation experiments [57]. The United Nations Scientific Committee on Effects of Atomic Radiation (UNSCEAR) 1996 report provided the example of a booklice, *Psocoptera* [17]. In this respect, the smaller population sizes of not only insect herbivores but also other various terrestrial organisms in Chernobyl could suggest insufficient amounts of food available for the following organisms, although direct effects on these organisms cannot be excluded: spiders [54,55], cicadas [54], dragonflies [54,55], butterflies [54,55], grasshoppers [54,55], bark beetles [17,55], bumblebees [54,55,56], booklice [17], springtails [17], soil invertebrates [57,58], reptiles [56], birds [54,59], and mammals [56]. IAEA (1992) [16] suggested that vegetation changes through indirect effects of radiation exposure are more critical than direct effects in invertebrates.

### 5.2. Pollen-Mediated Indirect Effects

A version of the food mass-mediated effect is the pollen-mediated effect, in which the reproductive and pollination systems of plants are specifically affected via direct irradiation. Pollens are foods for some insects, but the relationships between plants and pollinating insects (i.e., bees, butterflies, and others) are more complex than the simple predator-prey relationship. A decrease in the plant population may occur slowly through low pollen viability, resulting in a decrease in pollinating and other related insects. According to Grodzinsky and Gudkov (2006) [149] (cited in Geras’kin et al. (2008) [11]), an increase of nearly 30% in nonvital pollen was found in clover *Trifolium repens*, fireweed *Chamaenerium angustifolium*, and silene *Melandrium album* within 30 km of the Chernobyl NPP. In addition, pollen viability was negatively correlated with radiation intensity in 94 species of angiosperms during 1992–1994 (in *Viola matutina* during 1987–1988) and in 111 species during 2008–2011 [150].

Interestingly, Kim et al. (2019) [151] tracked the movement of the endophyte *Streptomyces globisporus* from strawberry plants to its pollinator, honeybees. This endophyte colonizes both strawberry flowers and the honeybee gut, revealing that honeybees play a role in transferring bacteria among strawberry flowers. More importantly, gray mold disease occurred in 42% of strawberry flowers without bacteria but in 12% of those with bacteria. In the case of honeybees, the mortality rates induced by the entomopathogens *Paenibacillus* and *Serratia marcescens* were over 70% without bacteria but 28% with bacteria. As demonstrated here, plant-associated microbes, plants, and pollinators are inseparably connected with each other. The microbiomes of plants and bees have similar functions and are dominated by similar taxa despite their different relative abundances [152]. Therefore, it is not unrealistic that the reduced pollen viability observed in Chernobyl disturbed their mutualistic interactions and led to fatal circumstances for survival. Regarding insect-pollinated fruit plants in Chernobyl, Møller et al. (2012) [153] statistically described that a decrease in pollinating insects resulted in decreased fruit production, decreased fruit-eating birds such as thrushes and warblers, and limited seed dispersal. Pollen degradation could also trigger similar negative interactions.

### 5.3. Metabolite-Mediated Indirect Effect

**Figure 3:**
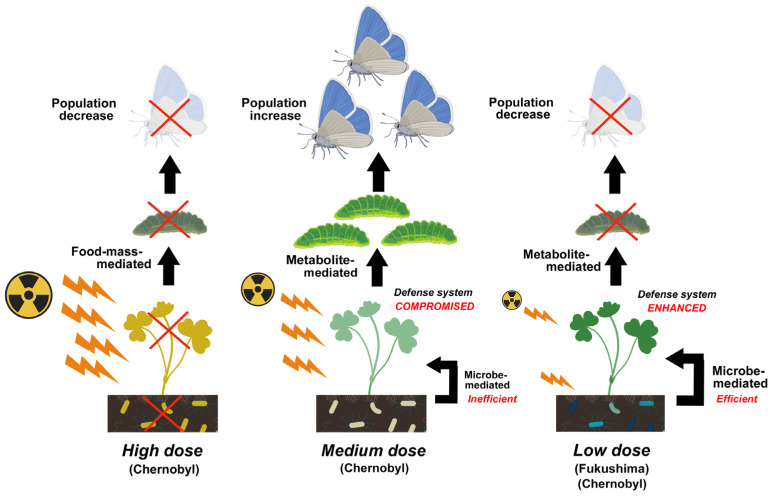
Comparison of possible mechanisms of population changes in insect herbivores among high-dose exposure (Chernobyl), medium-dose exposure (Chernobyl), and low-dose exposure (Fukushima and Chernobyl). In this example, both high-dose and low-dose pathways potentially cause a decrease in the population of adult butterflies in the field, but the medium-dose pathway causes the opposite result. This figure was created with BioRender.com and Adobe Photoshop Elements.

In Fukushima, the pollution level was relatively low compared to that in Chernobyl. One of the main radionuclides detected when measured was ^137^Cs in both Chernobyl and Fukushima, and its released amount in Fukushima was estimated to be, at most, 40% of that in Chernobyl [154]. This is probably why plants in Fukushima seem to be healthy, at least to the naked eye; no deleterious effects on plants have been reported, although there are a few reports on morphological abnormalities [133,134,155]. In this sense, food mass-mediated indirect effects (Figure 3) may not occur in Fukushima between plants and insect herbivores. Pollination also does not seem to be affected much in Fukushima [156]. However, lower abundances of insects such as butterflies [54,157] and cicadas [54] have been reported along with an increasing radiation dose. They are insect herbivores. Furthermore, a decreased abundance of birds [158,159,160] and decreased reproductive performance of goshawk [161] have also been reported. Birds rely on both plants and insects for their performance. Although these bird cases might have resulted from food-mass-mediated effects, at least partially, these population changes in insects and birds can occur through a different mechanism from the food-mass-mediated mechanism (Figure 3). The accumulation of genetic mutations caused by initial acute exposure is another valid explanation, but it seems to be a partial explanation; morphological abnormalities in butterflies are heritable (transgenerational) but correctable in the next generation with noncontaminated food [31] (see Introduction). Therefore, the decrease in the population of insect herbivores in Fukushima may constitute field-based evidence for metabolite-mediated indirect effects, as discussed below. An important hint may be obtained from the following study: the leaves of *Cirsium arvense* infected with endophytic fungi isolated from this species affect the foliar feeding amount and growth of insects, depending on the degree of specialism of insect herbivores [162]. As this example shows, biochemical changes due to infection in host plants could determine the population dynamics of insect herbivores.

Under radiation stress, plants respond well at the metabolic level (see also Section 4), which may be harmful (or advantageous) to insects. It is widely known that plants produce phytotoxins (secondary metabolites), and eaters have evolved to cope with phytotoxins [163,164]. Plant-associated microbes increase phytotoxin levels to improve the defense ability of host plants against insects under stress conditions [162,165]. Thus, metabolite changes maintain the delicate balance of phytotoxins in plants and plant tolerance in insects, and such changes are mediated by plant-associated microbes. It should be noted that in Fukushima, plant-associated microbes seem to be enhanced in contrast to those in Chernobyl (see also Section 4.3). Soil microbes may also be stimulated by low-dose radiation (see also Section 3.1), although such evidence in Fukushima has been lacking (see also Section 3.2 and Section 3.3).

Morita et al. (2022) [166] performed a feeding experiment with larvae of the pale grass blue butterfly *Zizeeria maha* using an artificial diet containing the antimicrobial agent ikarugamycin [167]. This metabolic compound was likely produced by endophytic bacteria in *Oxalis* leaves and was upregulated by irradiation (34 μGy/h for seven days) according to the LC-MS analysis [128]. Somewhat surprisingly, the pupation rate and eclosion rate significantly increased in response to ikarugamycin ingestion, indicating mild drug efficacy instead of toxicity. The authors speculated that ikarugamycin inhibited fungal and bacterial growth in the diet according to its antimicrobial efficacy [167,168,169]; hence, it could protect plants from pathogens in the field. These experiments suggest the possible contribution of metabolites from plant-associated microbes to the developmental process of insect herbivores in radioactively contaminated environments. Furthermore, butterfly larvae were also fed, in one study, an artificial diet containing lauric acid [166], a secondary metabolite of the host plant upregulated in response to radiation exposure [128]. Lauric acid had a mild toxic effect on larvae. In other words, lauric acid is a radiation-effect mediator from the host plant to the butterfly. Therefore, that particular study directly demonstrated a metabolite-mediated indirect effect on an insect herbivore.

In contrast, the plant defense system seems to be compromised at the medium-level exposure, probably due to its inability to synthesize phytotoxins and other metabolites at sufficient levels. As a result, insect herbivores may increase in population (Figure 3). This mechanism may also be considered the metabolite-mediated effect in a broad sense. It is known that forest and fruit trees within 30 km of the Chernobyl NPP were seriously injured by pests [170]. In irradiation experiments in the field, a population explosion of the aphid *Myzocallis discolor* occurred when white oak *Quercus alba* was exposed to 9.5 R/day (approximately 0.083 Gy/day) for 18 months [171], and xylophagous bark beetles invaded irradiated and weakened pine trees [17,57]. One of the candidates involved in the defense system is actinomyces, which produces chitinase [109]. In this way, actinomyces protects host plants from insect herbivores and pathogen invasion (see also Section 4).

The metabolite-mediated effect could occur in any herbivorous animals beyond insects, at least theoretically. It has been reported that the wild Japanese monkey *Macaca fuscata* in Fukushima shows hematological [172,173] and morphological [174] abnormalities and fetal growth delays [175]. These abnormalities may be caused by the direct exposure effect due to continuous exposure throughout their life span. However, they may be caused by the metabolite-mediated effects via plant foods, at least partially, if a wide variety of plants enhanced plant toxins in response to long-term low-dose exposure in Fukushima.

## 6. Conclusions

It is said that 10–100 billion bacteria and a large number of fungi and other microbes inhabit one gram of soil [107] and interact with each other in the soil ecosystem, responding sensitively to multiple stressors in the field. We must appreciate this vastness and flexibility. The accumulation of ^137^Cs and other radionuclides in soil appears to cause changes in the number, composition, and species diversity of soil microbes. Plant-associated microbes also appear to respond to radiation exposure. These changes certainly affect plant physiology, which seem to be inevitable, even at relatively low levels of radioactive pollution, because the sensitivity of microbes to radioactive pollution varies greatly depending on the species and environmental conditions. Importantly, such changes seem to persist for many years after a pollution event. Plants respond to these changes actively or passively, depending on the radiation level. The defense system of plants is likely enhanced at the low-level exposure, which may cause the eradication of insect herbivores in the field. The defense system of plants is compromised at the medium-level exposure, which may cause an increase in insect herbivores in the field. In any case, plant responses likely affect insect herbivores through food-mass-mediated, pollen-mediated, and metabolite-mediated interactions.

Although precisely distinguishing between direct and indirect effects requires many types of field surveys and laboratory experiments, because both effects work simultaneously in the field, indirect field effects are much less studied than direct effects but likely play a major role in the health of ecosystems in contaminated environments involving long-term low-dose radiation exposure. Population decreases in insect herbivores in Fukushima may be considered field-based evidence for metabolite-mediated indirect effects at relatively low contamination levels. We speculate that at the low exposure, the impacts of metabolite-mediated effects may be much greater than one might think, covering wide geographical areas and various species of insect herbivores in Fukushima. In this sense, the long-term impacts of microbe-plant interactions and metabolite-mediated interactions between plants and insect herbivores in the field cannot be overemphasized. These effects will then cause the adaptation of organisms to contaminated environments over time [20,176]. Epigenetic modifications, represented by DNA methylation, may occur as a mechanism of transgenerational effects [29,115,116,177,178,179]. Genetic changes at the population level may also be expected due to “natural” selection for more surviving individuals.

Many unknown types of field effects and many interesting cases of field effects will be discovered in the future, and their molecular mechanisms will be identified. Furthermore, the adaptation of organisms to contaminated environments over time will be clarified mechanistically at the molecular level. Such studies will help us to understand the whole picture of the biological and ecological impacts of the Chernobyl and Fukushima nuclear accidents.
